# Evaluation of performance for malaria diagnosis in health facilities by five provincial reference laboratories of China

**DOI:** 10.3389/fpubh.2023.1243642

**Published:** 2023-09-28

**Authors:** Xuan Zhang, Jingjing Jiang, Yuan Sui, Hui Yan, Jing Xia, Ying Liu, Lingcong Sun, Xiaoxiao Wang, Jutta Marfurt, Shenning Lu, Shizhu Li, Wei Ruan, Duoquan Wang

**Affiliations:** ^1^Zhejiang Provincial Center for Disease Control and Prevention, Hangzhou, China; ^2^Anhui Provincial Center for Disease Control and Prevention, Hefei, China; ^3^Brown School, Washington University, St. Louis, MO, United States; ^4^Guangxi Zhuang Autonomous Region Center for Disease Control and Prevention, Nanning, China; ^5^Hubei Provincial Center for Disease Control and Prevention, Wuhan, China; ^6^Henan Provincial Center for Disease Control and Prevention, Zhengzhou, China; ^7^Global and Tropical Health Division, Menzies School of Health Research, Charles Darwin University, Darwin, NT, Australia; ^8^College of Medicine and Public Health, Flinders University, Darwin, NT, Australia; ^9^National Institute of Parasitic Diseases, Chinese Center for Disease Control and Prevention (Chinese Center for Tropical Diseases Research), NHC Key Laboratory of Parasite and Vector Biology, WHO Collaborating Center for Tropical Diseases, National Center for International Research on Tropical Diseases, Shanghai, China

**Keywords:** malaria, laboratory diagnosis, accuracy, reference laboratory, China

## Abstract

**Introduction:**

The provincial malaria diagnosis reference laboratories review and assess malaria cases diagnosed in health facilities for supporting the malaria elimination efforts and preventing re-transmission of imported malaria. The study aimed to evaluate the detection capability of malaria diagnosis in China from 2014 to 2021.

**Methods:**

Data on malaria cases reported in the provincial-level administrative divisions (PLADs) of Anhui, Henan, Hubei, Guangxi, and Zhejiang from 2014 to 2021 were collected and analyzed.

**Results:**

In total, 5,770 malaria cases were reported from 2014 to 2021, and 99.05% (5,715/5,770) were submitted to the provincial malaria diagnosis reference laboratories. The median time between malaria cases being reported and the samples being received by reference laboratories was 6 days (Interquartile range, IQR:3–12 days) from 2017 to 2021. Diagnosis of 5,680 samples in the laboratory were confirmed by provincial reference laboratories, including 3,970 cases of *Plasmodium falciparum*, 414 of *P. vivax*, 1,055 of *P. ovale*, 158 of *P. malariae*, 1 of *P. knowlesi*, and 82 of mixed infections. *Plasmodium* species of 5,141 confirmed cases were consistent with the initial diagnosis, with a species accuracy rate of 90.53% (5,141/5,679). The accuracy of *P. falciparum* diagnosis in health facilities was higher than that of non-falciparum species. The inconsistency between microscopy and nested polymerase chain reaction (nPCR) results of confirmatory diagnosis was mainly in malaria-positive versus malaria-negative cases, as well as in mixed versus single infection cases.

**Conclusion:**

The provincial malaria diagnosis reference laboratories have played an important role in ensuring the accuracy and reliability of *Plasmodium* diagnosis in health facilities. However, the results of this study imply that capacity training for the identification of *Plasmodium* species in health facilities is warranted.

## Introduction

Malaria is a vector-borne disease caused by parasites of *Plasmodium* species that are transmitted to people through the bites of infected female *Anopheles* mosquitoes and is one of the major public health problems worldwide. In 2021, there were 247 million estimated cases of malaria worldwide, and 619,000 estimated malaria-related deaths. The World Health Organization (WHO) African Region carries a disproportionately high share of the global malaria burden (1). After decades of efforts, China has not reported indigenous malaria cases since 2017 (2) and was certified malaria-free by the WHO on the 30th of June 2021. However, a large number of imported malaria cases have been reported annually in China (3).

Timely detection and identification of all malaria cases, the first line of defense against malaria in China, is essential to prevent transmission and deaths (4, 5). Methods used for malaria diagnosis in health facilities include microscopy and rapid diagnostic tests (RDTs), and RDTs were usually based on the detection of *Plasmodium falciparum* lactate dehydrogenase (pf-LDH) and *Plasmodium* lactate dehydrogenase (pan-pLDH) antigens. Since 2011, the China malaria diagnosis reference laboratory network based on provincial Centers for Disease Control and Prevention (CDCs) and the Institute of Parasitic Diseases (IPDs) has been set up in stages to review and assess malaria cases using microscopy and molecular diagnostic tools (6). The provincial malaria diagnosis reference laboratories are primarily responsible for case reviews, malaria diagnostic capacity training, and quality assurance of the performance of malaria case identification in health facilities (7). A number of measures to control the internal quality assessment of malaria diagnosis, such as routine reviews of blood samples, and provincial blind sample assessment, have been carried out continuously. Furthermore, technique competitions of parasitic diseases including malaria were held to promote diagnostic capacity in health facilities in the province (8). Likewise, the provincial reference laboratory diagnosis provides guidance for epidemiological investigation and standardized treatment of reported cases.

Anhui, Henan, and Hubei provinces are historically malaria-endemic provinces in central China (9–11). Guangxi Zhuang Autonomous Region and Zhejiang Province, located in the south and southeast of China, respectively, are the regions with large number of imported malaria cases (12, 13). In order to assess malaria diagnosis and explore strategy to improve diagnostic capacity, we analyzed in this study the accuracy of malaria diagnosis in the five provincial-level administrative divisions (PLADs) of Anhui, Henan, Hubei, Guangxi, and Zhejiang from 2014 to 2021.

## Methods

### Case diagnosis and report

According to WHO malaria terminology, an imported case corresponds to a patient in which the parasite has been detected in a diagnostic test and malaria infection was acquired outside the area where it was diagnosed (14). Clinicians should order microscopic examination of thick and thin blood smears, as well as a rapid diagnostic test (RDT) for malaria. Malaria is a notifiable infectious disease in China, and health facilities at each of the administrative level are required to report cases to the China Information System for Disease Control and Prevention (CISDCP) within 24 h of malaria diagnosis.

### Reference laboratory confirmation

The CDC staff at the administrative county from where malaria cases were reported immediately conducted epidemiological investigation to identify the origins of malaria infection, as well as transported blood smears and samples to the provincial malaria diagnosis reference laboratories. Microscopic examination and nested polymerase chain reaction (nPCR) on each sample were performed by the provincial malaria diagnosis reference laboratories as described elsewhere (7, 15) to confirm the species, and the results of confirmatory diagnosis were subsequently fed back to county-level CDCs.

Criteria for confirmation of *Plasmodium* species in provincial reference laboratories: when *Plasmodium* was detected by microscopy only or nPCR only, the presence of *Plasmodium* was determined by using the results of the positive test method; when the results of microscopy and nPCR were inconsistent, the nPCR results overruled the results obtained by microscopy.

### Data source and collection

Data on malaria cases reported in the five PLADs from 2014 to 2021 were collected through the China Information System for Disease Control and Prevention (CISDCP) and the Information System for Parasitic Disease Control and Prevention (ISPDCP), a subsystem of CISDCP. Moreover, the date of samples received by the provincial reference laboratory has been more fully recorded since 2017. Epidemiological history, countries visited, date of illness onset, diagnostic institutions, date of report, microscopy and RDTs results reported by health facilities, microscopy and nPCR results confirmed by the provincial reference laboratories were used for analysis.

### Data analysis

Data were analyzed using Statistical Package for Social Sciences (SPSS version 25; SPSS Inc., Chicago). Box plot was used to describe time interval between cases being reported and samples being received. The accuracy rates of *Plasmodium* species was expressed by the consistency between *Plasmodium* species diagnosed in health facilities and *Plasmodium* species confirmed by the provincial reference laboratory.

## Results

### General description

A total of 5,770 malaria cases were reported from 2014 to 2021 (743 in Anhui, 1,643 in Guangxi, 1,285 in Zhejiang, 832 in Hubei, and 1,267 in Henan), all of which were imported malaria cases, with the exception of one case of infection through blood transfusion in Guangxi. The number of malaria cases ranged from 139 to 1,035 in the years studied. Most infections (5,537, 95.96%) originated in Africa. Specifically, 41.47% (2,393/5,770) of the cases in this study were imported from the West African Region, followed by countries in Central Africa (1,735, 30.07%) and Southern Africa (876, 15.18%). Ghana (14.80%, 854/5770), Nigeria (12.13%, 700/5770), Cameroon (9.79%, 565/5770), and Angola (9.58%, 553/5770) were the main importing countries. Cases from other countries of Asia accounted for 3.64% (210/5,770) of the total cases (Table 1).

**Table 1:** Origin of malarial infection in five PLADs from 2014 to 2021.

Origin of infection	The number (%) of cases	
	Number	%
Africa		
Central Africa	1735	30.07
West Africa	2,393	41.47
Southern Africa	876	15.18
East Africa	522	9.05
North Africa	9	0.16
Unknown countries in Africa	2	0.03
Asia	210	3.64
Oceania	14	0.24
South America	8	0.14
Blood transfusion	1	0.02

PLADs, provincial-level administrative divisions.

### Sample collection

**Figure 1:**
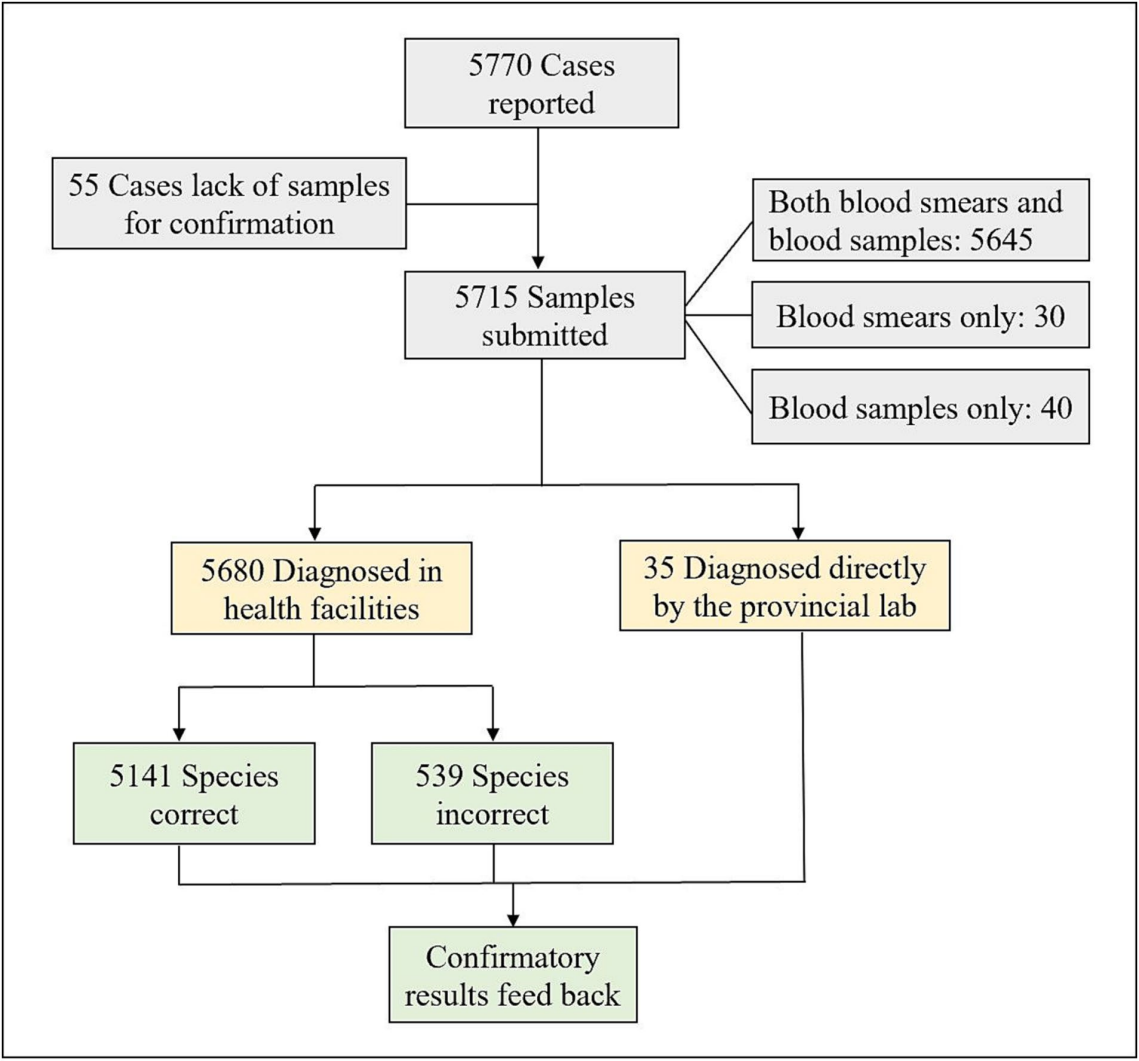
Flow chart of samples submitted to the reference laboratories for confirmation of malaria diagnosis in the five PLADs in China from 2014 to 2021.

Among the 5,770 reported malaria cases, 5,715 samples (5,715/5,770, 99.05%) were submitted to the provincial malaria diagnosis reference laboratories. The remaining 55 cases that lacked blood samples retained the initial diagnosis results of health facilities, mainly occurring between 2014 and 2016. Among the 5,715 submitted samples, 5,645 consisted of both blood smears and blood samples, 30 consisted of blood smears only, and 40 consisted of blood samples only. A total of 5,680 cases were initially diagnosed in health facilities using microscopy and RDTs, while the remaining 35 were directly diagnosed by the provincial laboratories. Figure 1 depict the flow chart of samples submitted to the provincial reference laboratories.

### Time interval between cases being reported and samples being received by the reference laboratories

**Figure 2:**
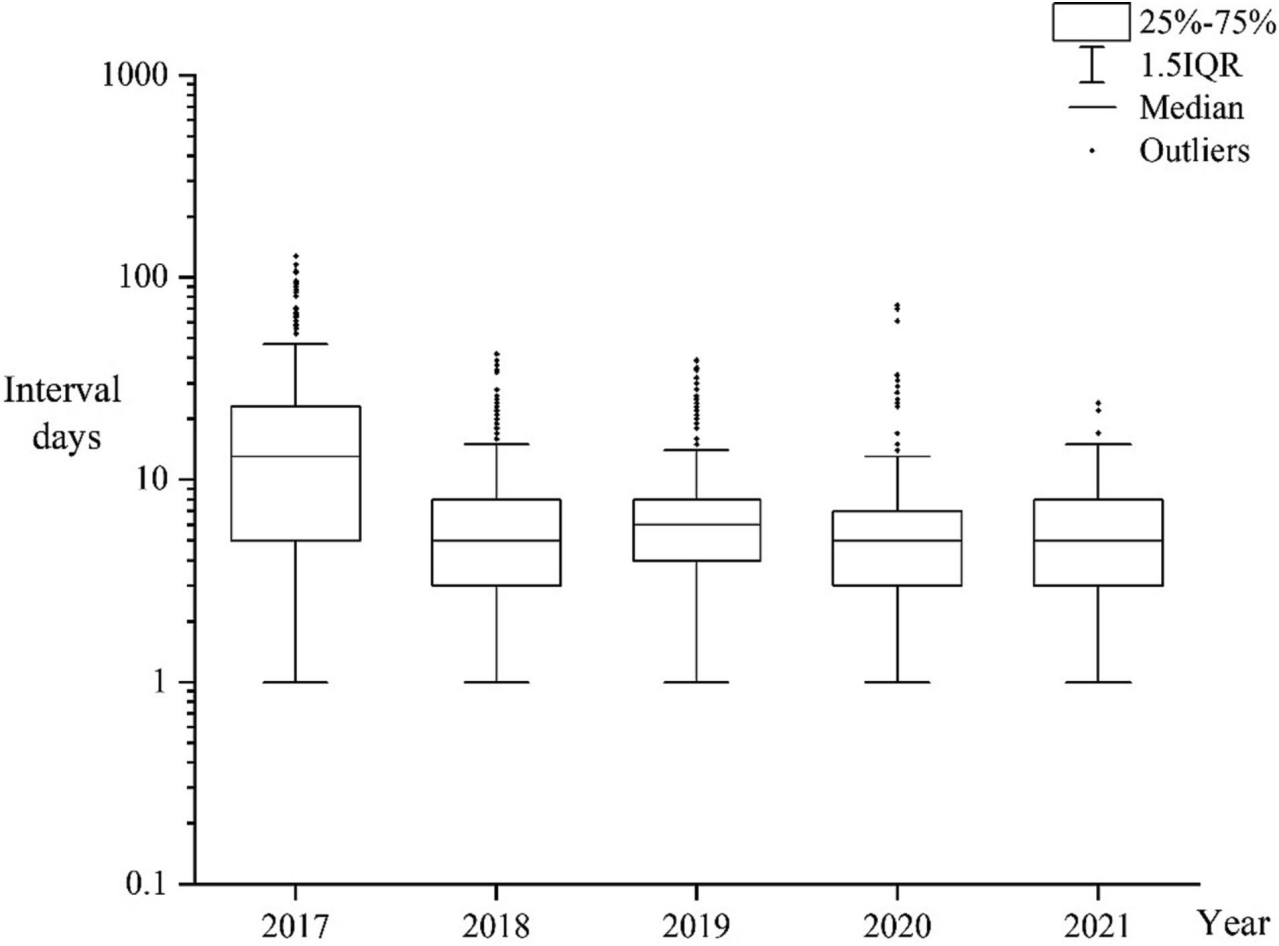
Box plot representing time interval between cases being reported and samples being received by the reference laboratories from 2017 to 2021.

From 2017 to 2021, the median interval between malaria cases being reported and samples being received by the provincial reference laboratories was 6 days (Interquartile range, IQR:3–12 days). Time interval in 2017 was longer than that in the 2018–2021 period (Figure 2). The interval for *Plasmodium falciparum* samples was longer than that for non-falciparum species samples, with the median interval being 7 (IQR:4–13 days) and 6 days (IQR:3–10 days), respectively. The interval for *Plasmodium vivax* samples was shorter than that for non-vivax species samples, with the median interval being 5 (IQR:3–9 days) and 7 days (IQR:4–13 days), respectively.

### Malaria cases initially diagnosed in health facilities

For the 5,680 malaria cases initially diagnosed in health facilities, results of 4,478 cases were obtained using both microscopy and RDTs. The malaria-positivity rates obtained by microscopy and RDTs at health facilities were 97.28% (4,356/4,478) and 92.50% (4,142/4,478), respectively. The consistency between the two methods was 89.77% (4,020/4,478) (Table 2).

**Table 2:** Malaria cases initially diagnosed using microscopy and RDTs from 2014 to 2021.

RDTs	Microscopic examination		Total
	Positive	Negative	
Positive	4,020	122	4,142
Negative	336	0	336
Total	4,356	122	4,478

### Results of confirmatory diagnosis obtained by reference laboratory

A total of 5,680 samples submitted to the provincial reference laboratories were initially diagnosed in health facilities, including 3,970 cases of *P. falciparum*, 414 cases of *P. vivax*, 1,055 cases of *P. ovale*, 158 cases of *P. malariae*, 82 cases with mixed *Plasmodium* species infections, and 1 case of *P. knowlesi*. The confirmation of *Plasmodium* species in 5,141 cases was consistent with the initial diagnosis (Figure 1), including 3,867 cases of *P. falciparum*, 370 cases of *P. vivax*, 769 cases of *P. ovale*, 102 cases of *P. malariae*, and 33 cases with mixed species infections, with an accuracy rate of 90.53% (5,141/5,679) (one case of *P. knowlesi* was not included; Table 3). The accuracy rates for species identification of *P. falciparum*, *P. vivax*, *P. ovale*, *P. malariae* and mixed infections were 97.41% (3,867/3,970), 89.37% (370/414), 72.89% (769/1,055), 64.56% (102/158), and 40.24% (33/82), respectively, and the accuracy of *P. falciparum* diagnosis was higher than that of non-falciparum cases. Comparison of species identification between the five PLADs showed that the accuracy of *Plasmodium* species identified in health facilities in Guangxi Zhuang Autonomous Region was higher than that in the other four provinces.

**Table 3:** Comparison of *Plasmodium* species initially diagnosed in the health facilities with those confirmed by the reference laboratories^*^.

PLAD	Confirmatory diagnosis	Initial diagnosis of *Plasmodium* species							
		Pf	Pv	Po	Pm	Mix	Unclassified^**^	Total	Consistency (%)
Anhui	Pf	526	2	0	0	0	7	535	526/535 (98.32)
	Pv	1	22	0	2	0	0	25	22/25 (88.00)
	Po	9	17	72	5	0	5	108	72/108 (66.67)
	Pm	4	7	1	16	0	0	28	16/28 (57.14)
	Mix	5	0	3	1	0	0	9	0/9 (0)
	Subtotal	545	48	76	24	0	12	705	636/705 (90.21)
Guangxi	Pf	963	4	1	1	10	2	981	963/981 (98.17)
	Pv	0	103	10	0	2	0	115	103/115 (89.57)
	Po	9	53	409	2	1	0	474	409/474 (86.29)
	Pm	2	2	2	21	0	0	27	21/27 (77.78)
	Mix	5	7	4	0	29	1	46	29/46 (63.04)
	Subtotal	979	169	426	24	42	3	1,643	1525/1643 (92.82)
Hubei	Pf	548	7	4	1	12	5	577	548/577 (94.97)
	Pv	7	99	1	0	0	1	108	99/108 (91.67)
	Po	8	20	79	3	1	3	114	79/114 (69.30)
	Pm	2	2	1	21	0	1	27	21/27 (77.78)
	Mix	0	0	1	0	2	0	3	2/3 (66.67)
	Subtotal	565	128	86	25	15	10	829	749/829 (90.35)
Zhejiang	Pf	930	2	2	0	6	9	949	930/949 (98.00)
	Pv	2	76	6	0	0	0	84	76/84 (90.48)
	Po	8	63	78	2	0	8	159	78/159 (49.06)
	Pm	2	5	5	20	0	1	33	20/33 (60.61)
	Mix	7	1	1	1	2	0	12	2/12 (16.67)
	Subtotal	949	147	92	23	8	18	1,237	1106/1237 (89.41)
Henan	Pf	900	6	2	2	3	15	928	900/928 (96.98)
	Pv	5	70	5	1	0	1	82	70/82 (85.37)
	Po	12	54	131	3	0	0	200	131/200 (65.50)
	Pm	8	8	3	24	0	0	43	24/43 (55.81)
	Mix	9	0	3	0	0	0	12	0/12 (0)
	Subtotal	934	138	144	30	3	16	1,265	1125/1265 (88.93)
Five PLADs	Pf	3,867	21	9	4	31	38	3,970	3867/3970 (97.41)
	Pv	15	370	22	3	2	2	414	370/414 (89.37)
	Po	46	207	769	15	2	16	1,055	769/1055 (72.89)
	Pm	18	24	12	102	0	2	158	102/158 (64.56)
	Mix	26	8	12	2	33	1	82	33/82 (40.24)
	Total	3,972	630	824	126	68	59	5,679	5141/5679 (90.53)

PLADs, provincial-level administrative divisions. ^*^ One case of *P. knowlesi* reported from Henan province was not included in this table. Pf, *Plasmodium falciparum*; Pv, *Plasmodium vivax*; Po, *Plasmodium ovale*; Pm, *Plasmodium malariae*; Mix, mixed *Plasmodium* species infection. ^**^ Unclassified by health facilities.

### Double confirmation of *plasmodium* species

Of the malaria samples submitted to reference laboratories, 5,645 cases were double confirmed using both microscopy and nPCR, 30 cases were confirmed by microscopy with blood smears only, and 40 cases were confirmed by nPCR with blood samples only. The consistency between microscopy and nPCR confirmation was 95.09% (5,368/5,645). Among the 277 inconsistent cases, 0.55% (31/5,645) were confirmed as malaria-positive using microscopy, but malaria-negative using nPCR, and 3.60% (203/5,645) were confirmed as malaria-negative using microscopy, but malaria-positive using nPCR. The remaining 43 cases (0.76%, 43/5645) were inconsistent in *Plasmodium* species identification, including 15 identified as *P. vivax* using microscopy versus *P. ovale* using nPCR, and 28 as single infections with *P. falciparum*, *P. vivax*, *P. ovale* or *P. malariae* using microscopy versus mixed infections using nPCR.

## Discussion

The malaria diagnosis reference laboratory network has provided technical support for identifying malaria cases during its elimination and prevention of re-transmission of imported malaria in China. Malaria diagnosis in China still faces many challenges, including improvement of microscopy competency at lower levels (7) and promotion awareness of malaria diagnosis in an elimination setting (16).

Microscopy is still the gold standard method for malaria diagnosis (17), although it is time-consuming and requires extensive technical experience. Malaria RDTs have been widely used in primary health facilities due to their simplicity and convenience (18). In fact, more than 78% of the total diagnoses were made using both microscopy and RDTs in the five PLADs, and microscopy showed better sensitivity than RDTs. Malaria RDTs are usually most effective in cases of *P. falciparum* (19). Jean et al. revealed that the sensitivity of RDTs was 93% (90.6–94.2%) for *P. vivax* in French Amazonia (20); whereas RDTs still showed low sensitivity and specificity for *P. ovale* and *P. malariae* in various other diagnostic contexts (21). In addition, malaria RDTs based on *P. falciparum* histidine-rich protein 2 (HRP2) raised malaria public health concerns because HRP2 deletions have been reported in different parts of the world (22). In view of their limitations, malaria RDTs are suggested to be complemented by microscopy to detect malaria, rather than being used as an alternative to microscopy in non-endemic areas (23, 24).

The accuracy for species identification initially diagnosed in the five PLADs was 90.51% from 2014 to 2021, which was higher than the 62–66% accuracy reported in China in 2015 (7), but similar to the 88.8% reported in Jiangsu Province in 2017 (25) and the 92.6% reported in Yunnan Province from 2013 to 2018 (26). Moreover, the accuracy of *P. falciparum* diagnosis remained above 94% in all five PLADs, but showed poorer accuracy for species identification of *P. ovale* and *P. malariae*. This may be due to the distribution of imported malaria species reported in China over recent years (27, 28), with *P. falciparum* as the predominant species, increased cases of *P. ovale*, and decreased cases of *P. vivax*. Consequently, the diagnostic capacity of health facilities for *P. falciparum* was maintained at a high level, whereas the capacity for non-falciparum species declined. Further comparison of species identification between the five PLADs showed that the accuracy of malaria diagnosis in Guangxi Zhuang Autonomous Region was higher than that in the other four provinces. This may be related to migrant workers returning from Africa (i.e., the Republic of Ghana) to Shanglin County of Guangxi in clusters which resulted in a significant increase in the number of malaria cases in 2013 (12). Therefore, the high prevalence of malaria cases at these might be advantageous in maintaining the microscopic ability to identify malaria in a stable state.

Case detection and accurate identification of *Plasmodium* species directly affect the implementation of malaria prevention and control measures (29). According to the national technical program for malaria elimination, the provincial reference laboratories for malaria diagnosis are required to provide timely feedback on results of confirmatory diagnosis to the administrative county from where malaria cases were reported. The results showed that the time interval from cases being reported to samples being received by the reference laboratories was shorter in 2018 to 2021 than that in 2017. Given the risk of local transmission caused by *P. vivax* (30) due to suitable vectors and conditions in historically malaria-endemic areas in China, timely detection of *P. vivax* for foci investigation and disposal to prevent re-transmission is of crucial importance. As shown in this study, the time interval was shorter for *P. vivax* than that for non-vivax species. Another reason to pay attention to *P. vivax* is that *P. vivax* forms dormant in the liver (hypnozoites) that can cause relapses and requires primaquine for radical cure (31), which remains a challenge to diagnosis and treatment.

The inconsistency between microscopy and nPCR confirmation at provincial reference laboratories was mainly in determining malaria positivity or negativity. Explanations for this included low parasite density affecting the ability to detect malaria by microscopy (32), some patients with illness history self-medicated after their return from overseas resulting in low detectability of viable parasites in the bloodstream by microscopy, or non-optimal sampling time of blood smears and samples for diagnosis (33). In addition, some differences in reference laboratory confirmatory diagnosis showed inconsistencies between mixed and single infections, as well as between *P. ovale* and *P. vivax*. Therefore, it is necessary to strengthen training to distinguish the morphology of *Plasmodium* species mentioned above and improve the detection ability of microscopists in samples with low parasitemia.

Our study has two main limitations. First, the study was retrospective; hence, some information may have gone missing during the study period, such as the time interval of samples received by the reference laboratories from 2014 to 2016. Secondly, the database only included reported malaria-positive cases; those initially diagnosed as malaria with confirmed malaria-negative results were not included in the analysis.

## Conclusion

The findings of this study showed that the accuracy of species identification by health facilities was higher than 90% in the five PLADs from 2014 to 2021, but fell short in the detection of *P. ovale and P. malariae.* Capacity training for the identification of *Plasmodium* species in health facilities needs to be strengthened. Multiple test approaches including microscopy and nPCR have enabled correct diagnosis of malaria, thus contributing to malaria elimination in China. The provincial malaria diagnosis reference laboratories have played an important role in ensuring the accuracy and reliability of malaria diagnosis in the elimination phase, as well as in the consolidation phase of malaria elimination.

## Data availability statement

The original contributions presented in the study are included in the article/supplementary material, further inquiries can be directed to the corresponding authors.

## Ethics statement

Ethical approval was not required for the study involving humans in accordance with the local legislation and institutional requirements. Written informed consent to participate in this study was not required from the participants or the participants’ legal guardians/next of kin in accordance with the national legislation and the institutional requirements.

## Author contributions

SZL, WR, and DQW conceived and designed the study. JJJ, YS, HY, JX, YL, LCS, XXW, and SNL contributed to data collection, finding interpretation, and the writing. XZ and JM performed the analyses. XZ wrote the manuscript. All authors contributed to the article and approved the submitted version.

## Abbreviations

WHO: World Health Organization
CDCs: Centers for Disease Control and Prevention
IPDs: Institute of Parasitic Diseases
PLADs: Provincial-level administrative divisions
RDTs: Rapid Diagnostic Tests
nPCR: Nested polymerase chain reaction
CISDCP: China Information System for Disease Control and Prevention
ISPDCP: Information System for Parasitic Disease Control and Prevention
IQR: Interquartile range
HRP2: Histidine-rich protein 2
